# Ethanol Impairs NRF2/Antioxidant and Growth Signaling in the Intact Placenta In Vivo and in Human Trophoblasts

**DOI:** 10.3390/biom9110669

**Published:** 2019-10-30

**Authors:** Sambantham Shanmugam, Dhyanesh Patel, John M. Wolpert, Caezaan Keshvani, Xiaobo Liu, Susan E. Bergeson, Srivatsan Kidambi, Lenin Mahimainathan, George I. Henderson, Madhusudhanan Narasimhan

**Affiliations:** 1 Department of Pharmacology and Neuroscience, Texas Tech University Health Sciences Center (TTUHSC), Lubbock, TX 79430, USA; sambantham.shanmugam@ttuhsc.edu (S.S.); dr.dhyanesh@gmail.com (D.P.); john.m.wolpert@ttu.edu (J.M.W.); caezaan.keshvani@ttuhsc.edu (C.K.); xiaobo.liu@ttuhsc.edu (X.L.); susan.bergeson@ttuhsc.edu (S.E.B.); george.henderson@ttuhsc.edu (G.I.H.); 2 Department of Department of Chemical and Biomolecular Engineering, University of Nebraska, Lincoln, NE 68588, USA; skidambi2@unl.edu; 3 Department Pathology, University of Texas Southwestern Medical Center, Dallas, TX 75390, USA; lenin.mahimainathan@utsouthwestern.edu

**Keywords:** NRF2, placenta, ethanol, trophoblasts, prenatal alcohol, CYCLIN-D1, p21

## Abstract

NRF2 is a redox-sensitive transcription factor that depending on the duration or magnitude of the stress, either translocates to the nucleus (beneficial) or is degraded in the cytosol (harmful). However, the role of NRF2-based mechanism(s) under ethanol (E)-induced developmental toxicity in the placental context remains unknown. Here, we used a rat prenatal model of maternal alcohol stress consisting of intermittent ethanol vapor (IEV) daily from GD11 to GD20 with a 6 h ON/18 h OFF in a vapor chamber and in vitro placental model consisting of HTR-8 trophoblasts exposed to 86 mM of E for either 24 h or 48 h. The role of NRF2 was evaluated through the NRF2-transactivation reporter assay, qRT-PCR, and Western blotting for NRF2 and cell growth-promoting protein, and cell proliferation assay. In utero and in vitro E decreased the nuclear NRF2 content and diminished its transactivation ability along with dysregulation of the proliferation indices, PCNA, CYCLIN-D1, and p21. This was associated with a ~50% reduction in cell proliferation in vitro in trophoblasts. Interestingly, this was found to be partially rescued by ectopic *Nrf2* overexpression. These results indicate that ethanol-induced dysregulation of NRF2 coordinately regulates PCNA/CYCLIN-D1/p21 involving growth network, at least partially to set a stage for placental perturbations.

## 1. Introduction

Prenatal alcohol exposure is generally considered to be the major single cause of preventable birth defects and developmental anomalies [1,2]. The earliest descriptions of toxic effects of ethanol (E) on the human fetus and its postnatal development are “impaired growth”, altered brain morphology, craniofacial malformations, cardiac septal defects, and others [3,4,5,6]. Subsequent studies utilizing animal models to define this phenomenon consistently documented the growth perturbant response to in utero and early postnatal E exposures [7,8]. Thus, potentially central to alcohol-induced multifactorial fetotoxic responses are the placenta’s role in transferring nutrients and oxygen to the growing fetus and regulating the maternal–fetal interaction. This concept has been reinforced by a body of studies illustrating E-related damage to the survival (apoptotic death) and differentiation of placental cells that may be mechanistically connected to its altered morphology, function (transport and endocrine), stress signaling response (shift in redox and inflammatory balance), and increased risk of placental abruption [9,10,11,12,13]. Importantly, following any stress, cellular-level responses in the placenta, for instance, alterations in the placental gene expressions, placenta-derived diffusible factors (secretome) such as brain-derived neurotrophic factor (BDNF), insulin-like growth factor 1 & 2 (IGF1, IGF2), serotonin, dopamine, norepinephrine, hormones, as well as oxidative stress (OS) can alone or in combinations, biologically impact maternal physiology as well as fetal neurodevelopment processes [14,15,16,17,18,19]. Of note, alcohol exposure can provoke all the above cellular impacts. Relevant to the current studies, others and we have identified numerous E-related perturbations of cellular events/signaling pathways that underlie fetal brain and placental cell survival and function [12,13,20,21,22,23,24]. Thus, despite being an ephemeral organ, the placenta’s vital role in the adaptive signaling to program whole system development (fetal growth) renders it a multifunctional organ beyond a mere passive channel for bidirectional maternal/fetal exchange.

A healthy placenta is capable of generating significant base-line levels of reactive oxygen species (ROS) to execute specific physiological functions [25,26]. However, a myriad of exogenous causes of OS such as stress, inflammation, or xenobiotics, in the absence of genetic abnormalities have been shown to modify specific gene/molecular patterns or decrease the antioxidant capacity that can adversely amplify ROS levels and lead to placental dysfunction [9,12,13,16,27,28,29]. E-induced OS has been addressed as a compelling mechanism/target in the placenta and human placental villi [29], as previously demonstrated by us for liver and brain [20,21,30,31,32]. Notably, this is a setting where perturbations in the cell cycle, proliferation, migration and cell morphogenesis are impacted [4,13]. The result of such a structure-function alteration at the placental level due to in utero insult might hinder their response and predispose the fetus and mother to an immediate as well as lasting health consequences. Thus, understanding how specific placental cytoprotective signaling mechanisms are altered in response to alcohol could provide critical insight into means to regulate the in utero environment to prevent adverse impacts on fetal development.

Relevantly, several lines of evidence have established that the redox-dependent transcription factor NF-E2 p45-related factor 2 (*Nrf2*; gene name *Nfe2l2*) integrates signaling from multiple inputs, including growth factors, nutrient, oxygen, and energy levels and positively regulates the genes encoding proteins that govern cell growth, metabolism, redox homeostasis, drug/xenobiotic detoxification, DNA repair, etc. [33]. Others and we have previously shown that activation of *Nrf2*/antioxidant pathway is essential to prevent alcohol-induced oxidative damage and related redox homeostasis in different model systems [20,34,35,36]. Given a prominent role for oxidative and growth signaling perturbations in alcohol-induced developmental toxicity together with an incomplete understanding of origin(s) of placental mechanisms, we investigated whether alcohol disrupts the interplay between *Nrf2*/redox pathway and growth network genes in the placenta. Using both an in vivo rat prenatal model of maternal alcohol stress and a placental cell in vitro acute E exposure model, we found that in utero and in vitro E exposure dysregulated NRF2 antioxidant signaling. This was associated with a significant reduction in the expression of proliferation markers, PCNA and CYCLIN-D1. In contrast, p21waf1/cip1, a key interactor of PCNA and a cell cycle progression inhibitor was significantly increased by E. All of these events could be connected to inhibition of cell growth in vitro. Additionally, cDNA-mediated *Nrf2* overexpression partially rescued the E-impaired NRF2-transactivation ability and cell proliferation.

## 2. Materials and Methods 

### 2.1. Materials

RPMI medium, L-glutamine, penicillin-streptomycin, and trypsin-EDTA were obtained from Gibco (Grand Island, NY, USA). Fetal bovine serum (FBS) was obtained from Atlanta Biologicals (Lawrenceville, GA, USA). Plasmocin was purchased from Invivogen (San Diego, CA, USA). BCA Protein assay reagent, SuperSignal West Pico chemiluminescence kit, NuPAGE 4%–12% Bis-Tris gels, was bought from Thermofisher (Rockford, IL, USA) respectively. Polyvinylidene difluoride (PVDF) membrane, iScript cDNA synthesis kit, and iTaq universal probes supermix were from Biorad Laboratories (Hercules, CA, USA). TriZol was purchased from Invitrogen (Carlsbad, CA, USA). Antibodies for NRF2 (Abcam, Cambridge, MA, USA), CYCLIN D1 (Cell Signaling Technology, Beverly, MA, USA); PCNA, p21, KEAP1, LAMIN-B1, H3, and GAPDH (Santa Cruz Biotechnologies, Santa Cruz, CA, USA); and ACTIN were from Sigma-Aldrich (St. Louis, MO, USA). Horse anti-mouse IgG-HRP, goat anti-rabbit, and IgG-HRP were obtained from Cell Signaling Technology (Beverly, MA, USA). HyBlot CL autoradiography film was got from Denville Scientific (Metuchen, NJ, USA). Trans IT-20/20 DNA transfection agent was obtained from Mirus Bio (Madison, WI, USA). TaqMan gene expression assays consisting of the primers and probes specific for different genes were from Applied Biosystems (Table 1) (Foster City, CA, USA). 2-2′7′ Dichlorofluorescin diacetate (DCF-DA), tertiary butylhydroquinone (t-BHQ), protease cocktail, 3-(4,5-Dimethyl-2-thiazolyl)-2,5-diphenyl-2H-tetrazolium bromide (MTT), and all other reagents were purchased from Sigma-Aldrich (St. Louis, MO, USA).

### 2.2. HTR-8 sv/neo Trophoblast Cultures and Ethanol Treatment

The immortalized extravillous trophoblasts line HTR-8/SVneo obtained from human placenta (ATCC, Manassas, VA. USA) was cultured in RPMI-1640 media containing 10% FBS supplemented with 200 mM l-glutamine, 100 Units/mL of penicillin/100 μg/mL streptomycin and plasmocin (5 µg/mL). Cells were grown in a monolayer at 37 °C in a humidified incubator with 95% air and 5% CO_2_. 24 h post-seeding the cells were either subjected to transfection experiments or E treatment. The concentration of E was 4 mg/mL (86 mM) a BAC that occurs in heavy drinkers and which elicits toxic responses in multiple animal models [20,37,38,39,40]. To maintain this ethanol level in culture media, the E-treated plates were incubated for 24 h or 48 h in an incubator pre-saturated with alcohol by placing a beaker filled with E for at least for 24 h [41,42], while the control cells were maintained in the normal incubator. These cells were shown to exhibit primary cytotrophoblast phenotypic markers (cytokeratin, human chorionic gonadotropin, and type IV collagenase) and exhibit response patterns similar to the former upon changing maternal environment [43,44]. Importantly, HTR-8/sv neo cells are regarded as an appropriate cell culture model to study normal trophoblast and placental biology as they are non-tumorigenic cells unlike the other widely used placental cell types, JEG3, BeWo, and JAR, which are choriocarcinoma cells.

### 2.3. In Vivo Prenatal Alcohol Exposure Model

#### 2.3.1. Rationale for the Timeline Used

The timeline of alcohol exposure in this study was between gestational days (GD) 11–20, a second-trimester equivalent in humans. The rationale for using this timeline is the first step towards one of our long-term goals to understand the influence of placental changes on the neurodevelopmental process during prenatal alcohol exposure. In particular, gestational day (GD) 11–20 is an active phase of fetal CNS development including but not limited to histogenesis, neurogenesis, vasculogenesis, angiogenesis, proliferation and migration, and differentiation [45,46,47]. Importantly, in mice, only by GD10.5 (GD12 in rats according to Carnegie staging), the placenta is shown to acquire a structure that consists of a layer of trophoblast giant cells, spongiotrophoblast, and the inner labyrinth (equivalent to human villi and where nutrient transfer occurs), which then until term undergoes expansion leading to placental growth [48].

#### 2.3.2. In Vivo Prenatal Alcohol Exposure

**Figure 1:**
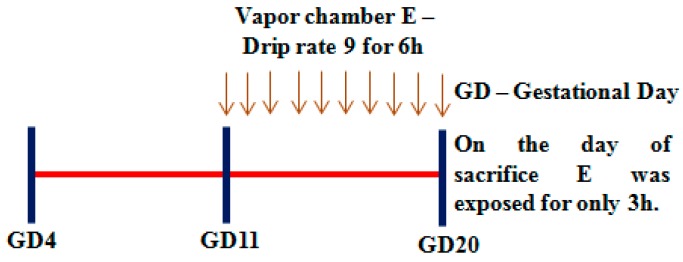
Experimental timeline of intrauterine ethanol exposure using vapor chamber.

The experiments utilized a well-established prenatal alcohol exposure animal model using vapor chamber (La Jolla Alcohol Research Inc, La Jolla, CA, USA) to achieve continuous low to moderate level blood alcohol level (BAL) with a slight modification [49,50]. This method not only causes a reliable increase in BAL, but is also less invasive with minimal handling and/or restraint stress influences. It is also much less labor intensive than other methods and multiple animals can be exposed simultaneously. Pregnant Sprague Dawley rats were divided into two experimental groups and subjected to the following regimens: (1) Experimental group (E)—This group received an intermittent 95% ethanol vapor (IEV) daily with a 6 h ON/18 h OFF in a vapor chamber starting gestational day (GD) 11 until GD 20 with the last exposure performed for 3 h before sacrifice to maintain blood alcohol levels. The E drip rate was set at 9 that fall into the heated flask to be evaporated and this was equivalent to approximately 90 mL/h [49]. The BAL measured by Analox AM1 analyzer was found between 160–185 mg/dL. (2) Control group (C)—This group of animals received ambient air exposure in place of ethanol vapor. The animals were subjected to experimental exposures during their light cycle from 10 AM–4 PM. The somatic symptoms such as gait, respiration, and locomotion were monitored once per 2 h during the exposure for high BAL-induced impairment. After 6 h exposure, the food pellets in the ethanol-exposed groups were removed and replaced with fresh pellets to avoid consumption of pellets with ethanol absorbed. Animals were handled only to measure the weight before and after the exposures. The schema of alcohol experimental regimen is illustrated in Figure 1. All animals were maintained by following the Institutional Animal Care and Use Committee-approved procedures (#16017). Both control and ethanol-exposed dams had full access to water ad libitum. At the end of treatment, dams were decapitated and blood was collected for alcohol analysis using Analox AM1 analyzer. Placentas were removed from the uterine horns and stored at −80 °C until use.

### 2.4. Transfection 

Transfection experiments were carried out as previously described [51,52]. Cells were either seeded in 6-well or 12-well plate at a density of 2.5 × 10^5^ and 1.5 × 10^5^ cells/well, respectively. The next day, cells were transfected with 200 ng of 4x antioxidant response element (ARE)-Luciferase reporter construct and 0.5 ng of Renilla reporter using TransIT 20/20. When *Nrf2* overexpression experiments were performed, 500 ng of pEF-Nrf2 or empty vector was co-transfected along with 4x ARE and renilla constructs. The construct and the transfection reagents were prepared separately using OPTI-MEM I media. Construct and the transfection reagent were mixed together and incubated for 20 min at room temperature to allow the formation of transfection complex. For the E treatment, 24 h after transfection of the respective constructs as mentioned above in Section 2.2, E was added to yield 4 mg/mL for additional 48 h.

### 2.5. Luciferase Assay

Transfection was performed in serum free, antibiotic free media as above (Section 2.4) and 1.5 h post-transfection media containing serum and antibiotics was added and the plates were returned to incubator. Following 24 h of transfection, E was exposed for additional 48 h. For the tBHQ experiments, 50 µM of tBHQ was added to cells when serum supplemented media was added post 1.5 h transfection. Twenty-four hours after tBHQ addition, E was added as above for the 48 h exposure. At the end of the experiments, luciferase reporter activity was assessed using dual-luciferase reporter assay system as per the manufacturer’s recommendation (Promega, Madison, WI, USA). The luciferase activity was measured using Glomax 20/20 luminometer (Promega, Madison, WI, USA) was normalized to renilla activity or protein concentration obtained from the corresponding samples.

### 2.6. RNA Extraction and Real-Time qRT-PCR Analysis

Total RNA was isolated from HTR-8 vs/neo cells or placental tissue using TRIzol reagent according to the manufacturer’s protocol (Invitrogen, Carlsbad, CA, USA). After eliminating the genomic DNA, 1.5 µg of total RNA was reverse transcribed using iScript cDNA synthesis kit (BioRad, Hercules, CA, USA). For the RT-PCR reaction, 1/10th of the cDNA was used in a final volume of 20 µL which contained 10 µL of iTaq Universal probes supermix, and 20 pmol of the respective primer/probe mix with the protocol initial denaturation step of 95 °C for 30 s which was followed by 40 PCR cycles (95 °C) for 5 s and 60 °C for 30 s. The relative expression of *Nrf2*, *Gclc*, *Gclm*, *Gsr*, *Ho-1*, *Nqo-1* was assessed by normalizing it to the housekeeping gene *Gapdh* and was then compared with controls. The relative fold variation in mRNA expression was calculated using the 2^−ΔΔCt^, where ΔCt = Ct _target gene_ − Ct _Gapdh_ and ΔΔCt = ΔCt _treated condition_ − ΔCt _untreated condition_.

### 2.7. Protein Extraction

#### 2.7.1. Whole Cell Extracts (WCE)

The whole cell protein extraction from HTR-8 sv/neo cells or placental tissues was prepared using radio-immunoprecipitation assay (RIPA) lysis as described [24]. Briefly, the cells or tissues were lysed or homogenized in RIPA buffer containing protease inhibitor cocktail at 4 °C. The lysates were sonicated for 5 s using vibra-cell ultrasonic processor (Sonics, Newtown, CT, USA) and centrifuged at 15,000× *g* for 25 min at 4 °C. The supernatant collected is the WCE.

#### 2.7.2. Cytosolic and Nuclear Extraction

Cytosolic and nuclear fractions from placental tissues were prepared as previously described [53]. One hundred mg of placental tissue was minced, and then homogenized on ice in STM buffer [250 mM sucrose, 50 mM Tris–HCl (pH 7.4), 5 mM MgCl2] which contained protease and phosphatase inhibitor cocktails. Homogenate was incubated on ice (30 min), vortexed twice at maximum speed (15 s) and centrifuged at 800× *g* for 15 min. The supernatant (S0) and pellet (P0) was used for cytosolic and nuclear fractions, respectively. S0 was spun at 800× *g* for 10 min and the resultant supernatant was spun at 11,000× *g* for 10 min. The collected supernatant containing cytosol and the microsomal fraction was then acetone precipitated at −20 °C for 2 h followed by centrifugation at 12,000× *g* for 5 min and the cytosolic pellet was resuspended in STM buffer. The pellet from the first step (P0) was resuspended in STM buffer, vortexed, spun twice at 500× *g* for 15 min and 1000× *g* for 15 min, and resuspended in NET buffer [20 mM HEPES (pH 7.9), 1.5 mM MgCl_2_, 0.5 M NaCl, 0.2 mM EDTA, 20% glycerol, 1% Triton-X-100, protease and phosphatase inhibitors]. This lysate was sonicated for 15 sec at 30% amplitude and centrifuged at 9000× *g* for 30 min at 4 °C. The supernatant was the final “nuclear fraction”. The cytosolic and nuclear extracts from HTR8/sv neo cells was prepared using a commercially available NE-PER Nuclear and Cytoplasmic Extraction kit (Thermoscientific, Rockford, IL, USA), respectively. The purity of cytosolic and nuclear fraction was determined by the expression of GAPDH and LAMIN-B1 respectively using immunoblotting.

### 2.8. Immunoblotting

Equal amounts of protein lysates were loaded on either NuPAGE 4%–12% Bis-Tris or 12% SDS gel and subjected to electrophoresis. The separated proteins were electro-transferred onto a PVDF membrane, rinsed in PBST, and non-specific binding was blocked with 5% non-fat dry milk powder in PBST for 1 h. Membranes were washed once in PBST for 3 min and probed with either one of the following primary antibodies against NRF2, PCNA, p21, CYCLIN-D1, KEAP1, LAMIN-B1, TUBULIN, H3, ACTIN, or GAPDH in PBST for 3 h or overnight at 4 °C as previously described [51,52] and subsequently washed with PBST three times. Later, the membranes were incubated with either anti-rabbit IgG or anti-mouse IgG-HRP secondary antibody conjugated with horseradish peroxidase in PBST (1:1000 or 1:5,000) for 1 h at room temperature. Washed blots (5 times × 5 min each) were then subjected to ECL-chemiluminescence detection of protein bands. The immunoreactive signals detected were captured onto an autoradiography film and scanned using Adobe Photoshop CS2 (v9.0, Mountain View, CA, USA). The intensity of target proteins was quantified using NIH Image J (Version 1.51K, Wayne Rasband, Bethesda, MD, USA) and normalized to either GAPDH or TUBULIN or ACTIN or LAMIN-B1 or H3 band intensity.

### 2.9. Reactive Oxygen Species Detection With DCF-DA Using Fluorimetry

DCF-DA staining was used for ROS detection with subsequent fluorimetry analysis [54]. Following the experiment, DCF-DA reagent (final concentration 25 μM/mL) was added to the cells and incubated in dark (37 °C) for 30 min in a 5% CO_2_ humidified incubator. Subsequently, cells were washed once, phenol red-free RPMI media added to replace the solution, fluorescence was measured immediately using GloMax Multidetection System (Promega, Madison, WI, USA). The excitation and emission were at 490 nm and 510–570 nm, respectively. Following the fluorescence measurement, the cells were trypsinized and resuspended in regular cell growth media and the viability was counted. The data was expressed the percentage changes in the ratio of DCF-DA fluorescence to 1 × 10^6^ living cells in the treated condition, after normalizing to untreated control cells (100% fluorescence/cell number).

### 2.10. MTT Assay

Cell viability was assessed using MTT assay. After completion of the experiment, followed by a gentle phenol red-free RPMI-1640 media wash, MTT at a final concentration of 0.5 mg/mL the cells were added and incubated at 37 °C for 2 h. The viable cells’ generated purple absorbance was recorded at 560 nm with 750 nm as reference wavelength using GloMax Multidetection System (Promega, Madison, WI, USA). The corrected absorbance values (560–750 nm) were expressed as the percentage of viable cells relative to that of untreated controls.

### 2.11. Statistical Analysis

Data are presented as mean ± S.E.M. When experiments involved more than two groups, one-way ANOVA followed by Student–Newman–Keuls post-hoc analysis was used to analyze the statistical differences. For experiments involving only two groups, Student’s *t*-test was used. The analysis was carried out using GraphPad Prism software (Version 5.02, San Diego, CA, USA). A *p* value less than 0.05 (< 0.05) was considered statistically significant.

## 3. Results

### 3.1. Prenatal IEV Exposure Did Not Alter the Placental and Body Weight

**Figure 2:**
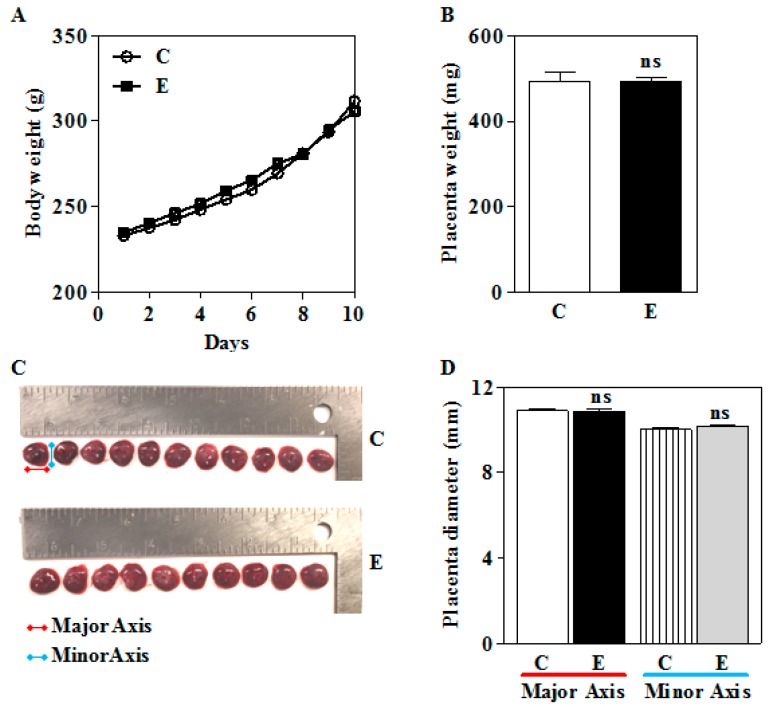
Effect of intrauterine alcohol exposure on gross body weight and placental changes. Pregnant rats (Sprague-Dawley) from gestational day 11 (GD 11) to GD20 were subjected to 6h ON/18h OFF everyday exposure of E with a drip rate of 9 or air (Control) by vapor chamber. (**A**) The gross body weight changes of the animals after everyday exposure of air (Control—C) and ethanol (E) exposure; (**B**) at GD20, a 3 h-exposure was used before the placenta was excised, trimmed off the membranes, washed serially in HBSS and PBS and the dry weight recorded (total n = 35 in C; n = 28 in E from 3 different litters); (**C**) representative placentas from a litter to show the size (total n = 35 in C; n = 28 in E from 3 different litters); (**D**); end-to-end placental diameter in millimeter (total n = 35 in C; n = 28 in E from 3 different litters). Values represent the mean ± SEM. ns—not significant vs. ethanol.

Previously, others and we have demonstrated that maternal ethanol exposure impairs placentation, growth, and placental function [10,13,29,55]. We initiated studies to address the effect of ethanol in vivo during second trimester equivalent in humans, which is an active phase of placental as well as fetal CNS development [46,47,48]. The chronic intermittent exposure (IEV) of alcohol was performed using vapor inhalation (Figure 1). This is a non-invasive procedure that has been shown to establish stable and consistent BAL besides having several advantages including but not limited to precise control of the dosing, duration, pattern of exposure, and overcoming handling and/or restraint stress-induced HPA influence, etc. [49,50]. Body weight remained unchanged upon E exposure (Figure 2A). The placental weight that serves as one of the standard measures of placental growth also remained unchanged in the study groups (Figure 2B). The placental shape (Figure 2C) and diameter (C vs. E, major axis: 10.89 ± 0.09 vs 10.88 ± 0.08; minor axis: 10.01 ± 0.08 vs 10.15 ± 0.04) (Figure 2D) also did not change significantly in response to E.

### 3.2. In Utero Ethanol Exposure Reduces Nuclear NRF2 Levels

**Figure 3:**
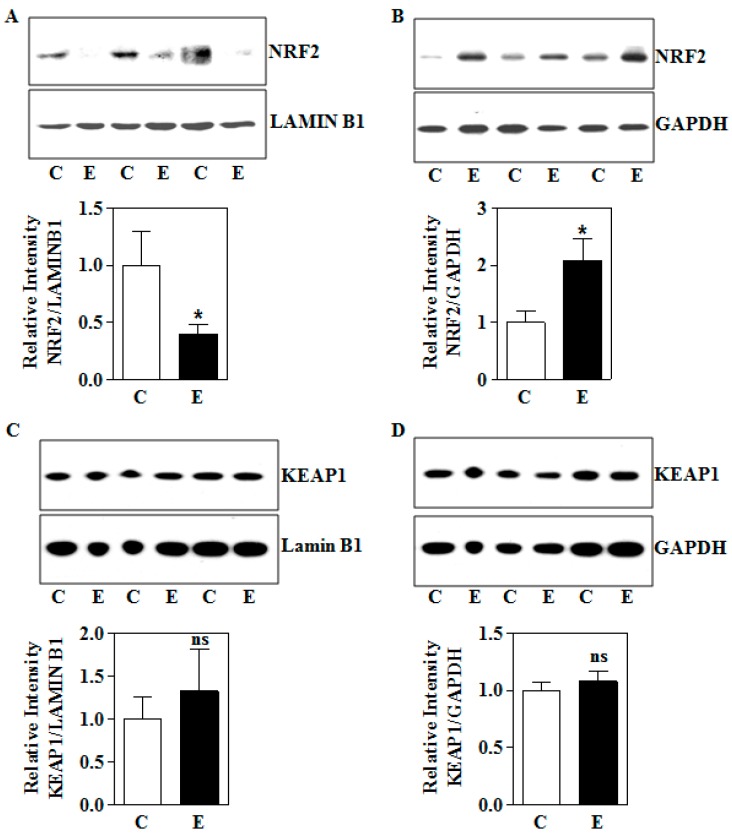
Effect of intrauterine alcohol exposure on NRF2 and KEAP1 protein expression. (**A**) A representative immunoblot image detecting placental NRF2 and LAMIN B1 protein expression in nuclear fraction (upper panel) and its quantification (bottom panel) from air (C) and IEV (E) exposed rats (hours) (total n = 12; n = 4 each from 3 different litters); (**B**) immunodetection of placental NRF2 and GAPDH protein in the cytosol of air (C) and IEV (E) exposed pregnant rats measured by Western blotting (upper panel) and the corresponding quantification of NRF2 protein levels normalized to the reference protein, GAPDH (bottom panel) (total n = 12; n = 4 each from 3 different litters); (**C**) Western blot image of KEAP1 and LAMIN B1 protein in placenta obtained from air (C) or vapor based ethanol (IEV)-exposed pregnant dams (upper panel) and the image densities of KEAP1 relative to LAMIN B1 (bottom panel) (total n = 12; n = 4 each from 3 different litters); (**D**) representative immunoblots demonstrating the expression of KEAP1 protein, with GAPDH as a loading control in placenta of control and intrauterine alcohol-exposed pregnant rats (upper panel) and the quantification of immunoblots of KEAP1 normalized to GAPDH (bottom panel). Values represent the mean ± SEM. * *p* < 0.05 was considered significant vs ethanol.

Prior studies have illustrated that a maternal two-day E binge model elicits a redox imbalance and oxidative damage to the fetal brain which causes enhanced apoptotic death of fetal cerebral cortical neurons. This occurs concomitantly with an upregulation of nuclear factor erythroid 2-related factor 2 (NRF2), which provides an important albeit incomplete protection from these events [20]. Since ethanol can also elicit oxidative damage in the placenta [13,29,55] and activation of NRF2 has been correlated with translocation of this protein into the nucleus, we first tested the nuclear levels of NRF2. In contrast to the fetal cerebral cortex setting, prenatal E exposure significantly decreased the nuclear levels of NRF2 (transcriptionally active) by 61% in the placenta (*p* < 0.05; Figure 3A,B). This was associated with an anti-parallel increase in the cytosolic retention of NRF2 (*p* < 0.05; Figure 3C,D). Keap1, an inhibitor and negative regulator of NRF2 that mediates both translocation to the nucleus and export from the nuclear compartment, remained unchanged (*p* < 0.05; Figure 3E,F). These results indicate that gestational E exposure reduces nuclear levels of NRF2 as well its translocation to the nucleus from cytosol likely in a Keap1-independent manner.

### 3.3. In Utero Ethanol Represses NRF2-Mediated Transcriptional Activation in the Placenta

**Figure 4:**
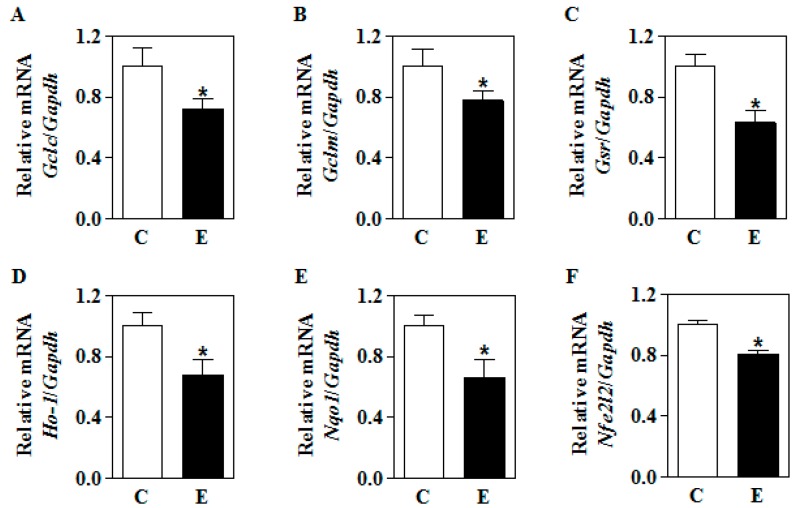
Effect of in utero alcohol exposure on the mRNA expression of Nfe2l2 and NRF2 target genes’ in the placenta. Pregnant SD rats were exposed to air (C) or IEV (E) for the periods indicated in Figure 1 and real-time qRT-PCR analysis for NRF2 target genes’ was performed. (**A**) The fold change mRNA expression of *Gclc*; (**B**) *Gclm*; (**C**) *Gsr*; (**D**) *Ho-1*; (**E**) *Nqo1*; and (**F**) *Nfe2l2* was determined by normalizing with the expression of a housekeeping gene, *Gapdh*. A total of 12 samples with 4 each from 3 different litters were examined. Values represent the mean ± SEM. * *p* < 0.05 was considered significant versus ethanol.

As in utero E decreased the placental nuclear NRF2 levels, we next determined whether this was associated with a decrease in some of its well-known downstream target genes expression. Multiplex Taqman real-time qRT-PCR analysis for glutamate–cysteine ligase catalytic subunit (*Gclc*; Figure 4A), glutamate–cysteine ligase modifier subunit (*Gclm*; Figure 4B), glutathione reductase (*Gsr*; Figure 4C), hemeoxygenase (*Ho-1*; Figure 4D), and NAD(P)H quinone dehydrogenase 1 (*Nqo1*; Figure 4E) using differentially labeled probe for individual genes (FAM probe) and *Gapdh* (VIC probe) showed a significant decrease in their transcript expression (*p* < 0.05). Interestingly, the mRNA expression of NF-E2 p45-related factor 2 (*Nfe2l2* or *Nrf2*) gene, which is known to undergo self-regulation was also found to be significantly repressed (*p* < 0.05) (Figure 4F). These data illustrate that in utero *E*-induced NRF2 reduction appears to functionally reflect an impairment of transcriptional activation of its target genes.

### 3.4. In Vitro E Impairs Nuclear Nrf2 Levels and its Transactivation Function in Human Trophoblasts

**Figure 5:**
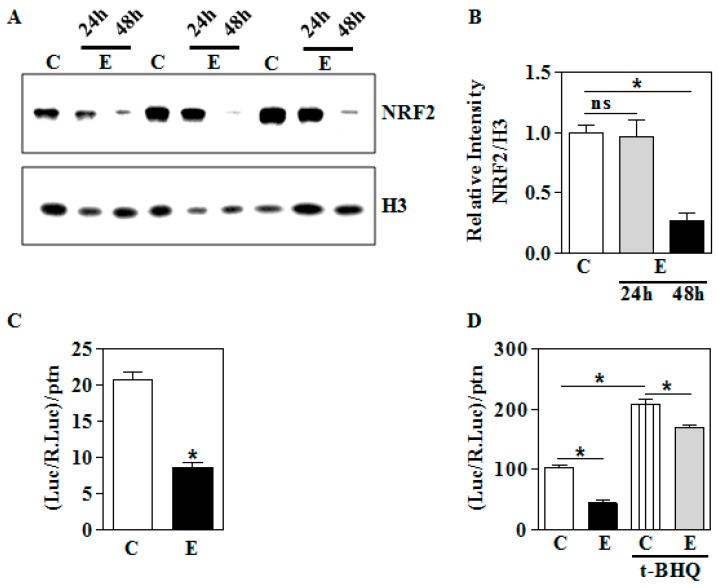
Effect of in vitro ethanol exposure on the nuclear levels of NRF2 and its transactivation ability. HTR8/sv neo trophoblasts were treated with E (4 mg/mL) as indicated in Section 2.2. (**A**) The protein levels of NRF2 in the nucleus were determined by immunoblot analyses and equal loading was assessed by anti-H3; (**B**) the densitometric scanning analysis ratio of NRF2 to H3 band intensities was performed using NIH ImageJ analysis; (**C**) cells were transfected with luciferase construct containing NRF2-binding ARE sequences with 4 tandem repeats along with pRL-renilla construct as detailed in Section 2.4. NRF2 transactivation was determined in terms of luciferase normalized to renilla and to protein (*n* = 12); (**D**) cells were transfected and processed as in (**C**) but for t-BHQ pretreatment for 24 h (*n* = 12). Values represent the mean ± SEM. * *p* < 0.05 was considered significant versus ethanol.

Having shown that gestational E impairs the NRF2 nuclear localization and its transactivation function in utero, we next tested whether there is an alteration in NRF2 and its activity in vitro following E exposure in human HTR8/sv neo trophoblast cells. Immunoblotting analyses indicated that 24 h did not significantly alter nuclear NRF2 content, while a significant diminution is seen at 48 h by about 70% at 48 h (*p* < 0.05; Figure 5A,B). Since we did not observe any change in nuclear NRF2 at 24 h, we measured the basal transactivation function of NRF2 in terms of 4x ARE-driven luciferase reporter activity. As expected, we observed a parallel reduction in 4x ARE-luciferase activity by about 60% indicating a functional correlation (*p* < 0.05; Figure 5C). Since NRF2 is a stress-inducible transcription factor, we induced NRF2 activity by tBHQ, and interestingly, found that E was able to reduce the inducible NRF2-dependent transactivation significantly (*p* < 0.05; Figure 5D). However, unlike basal condition, E was able to reduce the inducible ARE reporter activity only by approximately 20% (*p* < 0.05; Lane 3 vs. 4; Figure 5D). These results support our hypothesis that alcohol can perturb NRF2 and its function under physiological (basal) and inducible (overexpression) setting in human HTR8/sv neo trophoblasts.

### 3.5. In Vitro E Inhibits Cell Viability in Association with Dysregulation of PCNA, Cyclin D1, and p21 in Human Trophoblasts

**Figure 6:**
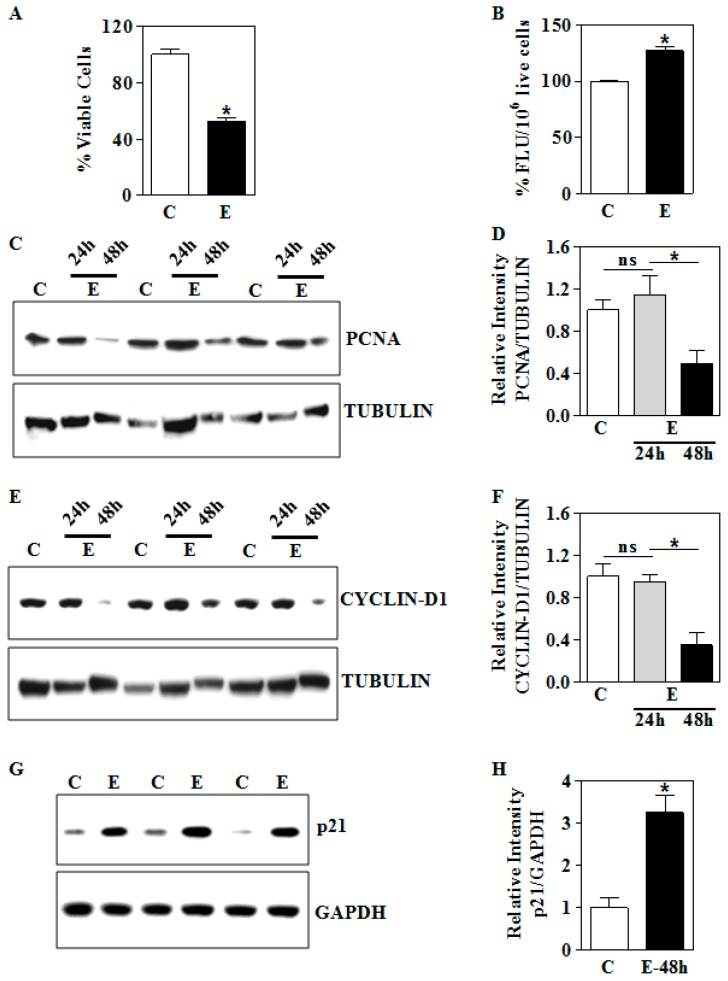
In vitro ethanol effects on the viability and expression of growth-related proteins in HTR8/sv neo trophoblasts. (**A**) E-treatment at a concentration of 4 mg/mL of E was done for 48 h. The trophoblasts cell viability was measured using MTT assay and shown as percentage viable cells; (**B**) effect of 48 h E on intracellular ROS level measured by fluorometry after DCF-DA treatment. The results are expressed as percentage fluorescence units (FLU) per the cell number; (**C**) equal amounts of proteins from E-treated trophoblasts at the indicated time points were resolved by SDS-PAGE and subjected to immunoblot directed against PCNA and a representative blots are shown; (**D**) graphical representation of the quantification of PCNA normalized to TUBULIN expression; (**E**) representative immunoblot for CYLCIN-D1 protein expression following E exposure; (**F**) semi-quantitative analysis of CYCLIN-D1 and TUBULIN ratios of experiments from (**E**); (**G**) immunoblot analysis for p21 protein expression following 48 h E exposure of trophoblasts and GAPDH blots served as loading control; (**H**) the densitometric quantification of p21 normalized to GAPDH levels. Values represent the mean ± SEM from n = 6. * *p* < 0.05 was considered significant versus ethanol and ns indicates not significant.

Having shown the dysregulation of NRF2 by E exposure, we next addressed the functional or phenotypic relevance of E-induced NRF2 loss. To this end, we assessed the growth of trophoblasts by MTT assay. Following E treatment, we observed growth-suppression of human trophoblasts by about 50% (*p* < 0.05; Figure 6A) relative to control at 48 h. This suggests that NRF2 loss has some association with the growth inhibition. Since E-inhibited NRF2 associated transactivation of antioxidant genes, it is reasonable to expect ROS levels to be enhanced. To this end, we measured the intracellular ROS level using DCFDA-based fluorescence. The fluorescence intensity of DCF-DA normalized to 1 million living cells was significantly increased in E treated cells by approximately 30% (*p* < 0.05; Figure 6B) in relation to control cells indicating that the reduced NRF2 in the cells was permissive for ROS accumulation. To dig deeper into the mechanism of E-induced cellular growth suppression, we next assessed the expression pattern of proliferating cell nuclear antigen (PCNA), since it is one of the critical determinants of growth and proliferation of cells. In line with the cell growth inhibition data, we found that 48 h E-significantly decreased the protein expression of PCNA by about 50% (*p* < 0.05; Figure 6C,D) in human trophoblasts. We next determined the expression of CYCLIN-D1, a key cell cycle protein required for the progression of the cell cycle from G1 to S that mediates growth. E-exposure significantly blunted the expression of CYCLIN D1 protein by 65% (*p* < 0.05; Figure 6E,F). Since p21 can simultaneously bind to the DNA polymerase delta processivity factor, PCNA, and CYCLIN-D1 and inhibit their growth-promoting function, we posited p21 levels may go up in response to E. Consistent with this, we found that E treatment significantly enhanced the p21 levels by three-fold when compared to control cells (*p* < 0.05; Figure 6G,H). This is an indication that p21 may collaborate with PCNA and CYCLIN-D1 to repress the growth of trophoblasts upon E.

### 3.6. In Utero E Affects the Expression of Growth Perpetuating Factors in Placenta

**Figure 7:**
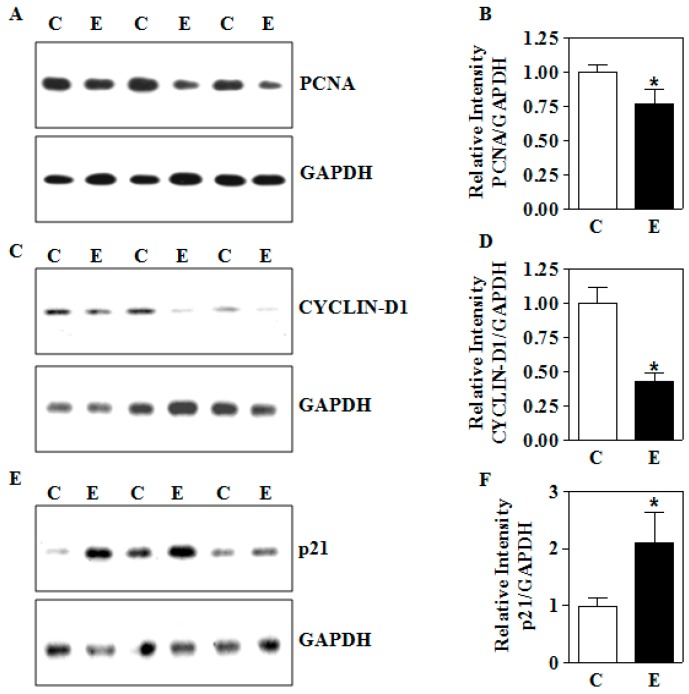
Effects of intrauterine IEV exposure on the protein expression of growth modulators in the placenta. The IEV exposure was performed as in Figure 1. (**A**) Representative immunoblots and (**B**) densitometric summary for PCNA in the placenta; (**C**) CYCLIN-D1 protein expression by immunoblotting and (**D**) its densitometric quantification in the placental tissue; (**E**) determination of placental p21 protein expression and (**F**) its quantification summary. For each measurement, a total of 12 placentas with 4 each from 3 different litters were used. Values represent the mean ± SEM. * *p* < 0.05 was considered significant versus ethanol.

Next, we determined whether the E-induced in vitro expression changes in growth-related factors observed in human trophoblasts are replicated in placentas from in utero E-exposed animals. Akin to the in vitro changes, maternal E exposure significantly abolished the expression of PCNA by about 30% (*p* < 0.05; Figure 7A,B) and CYCLIN-D1 by about 55% (*p* < 0.05; Figure 7C,D) in the placenta. Since an increase in p21 is correlated to growth arrest, we then analyzed p21 expression under the same conditions. In contrast to PCNA and CYCLIN-D1 expression, p21 protein expression was significantly upregulated by ~2.1-fold (*p* < 0.05; Figure 7E,F). These results illustrate that gestational E can impair the growth-promoting PCNA/CYCLIN-D1/p21 network.

### 3.7. Genetic Overexpression of Nrf2 Partially Ameliorates the E-Induced Growth Suppression Phenotype

**Figure 8:**
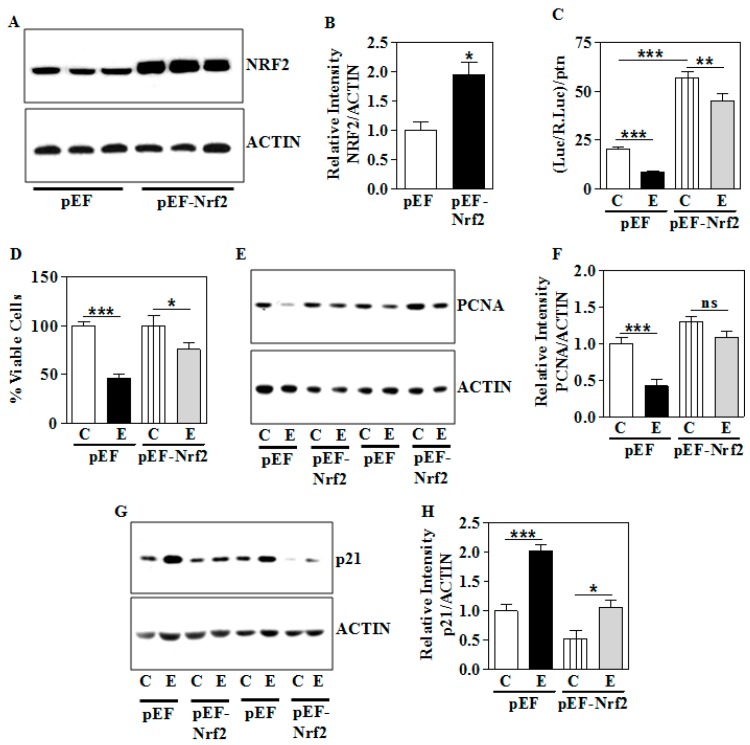
Effect of overexpression of *Nrf2* on the E-induced dysregulation of NRF2, growth modulators, and cell proliferation. The E exposure was performed as in Section 2.2. (**A**) The presence of overexpressed NRF2 was verified by immunoblotting in the WCE (Lanes 1–3: Empty vector; Lanes 4–6: *Nrf2* cDNA); (**B**) densitometric analysis of NRF2 protein normalized to ACTIN after *Nrf2* overexpression; (**C**) luciferase activity of the NRF2-responsive repeats of antioxidant responsive element in cells co-transfected with either empty or *Nrf2* construct as in Section 2.4 and treated with E for 48 h as in Section 2.2. The values shown indicate the ratio of the luciferase activities normalized to renilla and protein; (**D**) transfection was performed as in (**C**) and MTT assay was performed as in Section 2.10 and the results are expressed as percentage change from control; (**E**) Western blots with anti-PCNA on lysates of empty vector (pEF) or *Nrf2* cDNA (pEF-*Nrf2*) overexpressed human HTR8/sv neo trophoblasts. Anti-ACTIN was used as a loading control; (**F**) relative levels of the PCNA normalized to ACTIN quantified by NIH Image J analysis; (**G**) Gel images showing p21 protein expression and ACTIN as a loading control in the empty vector (pEF) or *Nrf2* cDNA (pEF-*Nrf2*) overexpressed human trophoblasts; (**H**) Quantification of p21 band intensity normalized to ACTIN intensity from Panel E. Values represent the mean ± SEM from n = 6 (**A**), (**E**), (**G**) and n = 12 (**C**) and (**D**). * *p* < 0.05, ** *p* < 0.01, **** *p* < 0.0001 vs. indicated group via Student–Newman–Keuls post-hoc analysis following one-way ANOVA. ns indicates not significant.

Finally, to confirm whether the gain of function of NRF2 rescues E-induced growth inhibition of trophoblasts, we performed exogenous overexpression of Nrf2 using a cDNA construct. The efficiency of cDNA-mediated Nrf2 overexpression was confirmed by a significant increase in the levels of NRF2 protein in WCE (*p* < 0.05; Figure 8A,B). Having verified the expression of NRF2, we next assessed the effect of E-induced NRF2-dependent transactivation repression with and without Nrf2 overexpression. Empty vector-transfected cells, when exposed to E, showed significant repression of 4x ARE reporter activity by about 58% (*p* < 0.05; Lane 1 vs. 2, Figure 8C). However, overexpression of the Nrf2 construct led to significant improvement of E-induced loss of transactivation ability (Figure 8C). In particular, E was able to reduce the inducible ARE reporter activity only by 21% (*p* < 0.05; Lane 3 vs. 4; Figure 8C). In parallel, E repressed the percentage of viable cells significantly by about 55% in the empty vector-transfected cells (*p* < 0.05; Lane 1 vs. 2, Figure 8D). However, when Nrf2 was overexpressed the survival percentage was promoted and E-induced decrease in survival was only around 25% (*p* < 0.05; Lane 3 vs. 4, Figure 8D). This indicates that Nrf2 overexpression appears to partially prevent the E-induced growth repression of trophoblasts. Further, E-induced PCNA silencing was significantly restored upon ectopic overexpression of Nrf2 (*p* < 0.05; Figure 8E,F). Conversely, Nrf2 overexpression in trophoblast cells elicited a normalization of E-induced p21 levels towards control (*p* < 0.05; Figure 8G,H). These data show that restoration of NRF2 signaling could play a pivotal role in regulating the E-induced reduction in cell growth, at least, partially.

## 4. Discussion

An intact placenta and functional placental cells are vital for normal fetal growth and survival. Our study demonstrates that maternal IEV stress elicits impairment in the master redox regulator, NRF2 in association with modulation of growth-related parameters in rat placenta and human trophoblasts exposed to E. The principal findings of the current study include: (1) Placentas from pregnant rats exposed to IEV displayed a blunted NRF2 and its transactivation function, which was also captured in the in vitro exposure of E to HTR8/sv neo, a human trophoblast line; (2) E-induced dysregulation of NRF2 signaling is associated with a reduction in the expression of growth-promoting factors, PCNA and CYCLIN-D1 both in vivo and in vitro; (3) These changes were accompanied by upregulation of cyclin inhibitory protein p21; and (4) An ectopic overexpression of *Nrf2* rendered partial recovery of E-induced dysregulation of growth-promoting factors and cell survival of human trophoblasts. Together, these results illustrate that downstream of E-related NRF2 regression, prenatal maternal IEV stress aberrantly regulates the growth effectors such as PCNA, CYCLIN-D1, and p21.

It is to be noted that many of the signaling changes assessed in the in vitro trophoblasts exposed to E in the present study were replicated in vivo in rat placenta tissue. First, this is in keeping with the data that both in vitro and in utero E exposure elicited a nuclear NRF2 diminution and its transactivation ability in trophoblasts and placental tissue, respectively (Figure 2 and Figure 3). This is a relevant finding because, although in utero E has been previously shown to impair redox homeostasis in which NRF2 was enhanced as an adaptive response in non-placental setting to at least partially counter the injury, this is the first demonstration that E-induces NRF2 downregulation in placenta in vivo following the maternal IEV exposure and also to trophoblasts in vitro. The disruption of redox balance with a retarded NRF2 function has been shown to cause placental dysfunction in several preclinical and in vitro E-mimicking oxidative stress inducing conditions such as cigarette smoke, fructose, and 5-α-dihydrotestosterone and insulin resistance [56,57,58]. Further, in a non-placental setting, we have previously found that persistent and/or severe oxidative stress can dampen NRF2 signaling via microRNAs (miR) such as miR-144, miR-142-5p, miR-153, and miR27a [51,52]. Another independent study identified miR142 and miR144 as pregnancy-associated miRs that were found to be involved in congenital malformations [59].

To further understand the impact of NRF2 impairment on the phenotypic outcome, we assessed the proliferation of trophoblasts in vitro as well as the gross placental and body weight as characteristics of growth. Typically, in the alcohol-vapor exposure model, during the first week or less, issues associated with a reduced rate of body weight gain in E-exposed animals is plausible. However, this has been circumvented by coordinating our alcohol exposure during the light phase of the light/dark cycle where animals are less active and less likely to consume food. This ensures that changes in food and water consumption pattern that normally occurs during the active (dark) phase do not influence our experimental condition. Data from this study indicated that in vitro E exposure while exerting growth suppression responses in human trophoblasts (Figure 6A,B) in tandem with a blunted NRF2 signaling, the in vivo IEV exposure had no significant effect on gross body or placental weight change (Figure 2). Consistent with our results, a previous finding in pregnant mice has demonstrated that alcohol exposure through vapor chamber for 4 h daily during GD5-20 displayed no discernible changes in the fetus weight and placenta weight [50]. However, another study has illustrated significant repression of placental growth, albeit, in a continuous liquid diet-based alcohol exposure model from GD6-18 in Long Evans rats [12]. The differences connected with the in vitro and in vivo data could be easily due to complex environmental differences in trophoblast milieu with effects of E on trophoblasts in vitro being direct while in the in vivo niche, such impacts could reflect multiple maternal-related factors. These could be interactions of other cell types (from the maternal system) such as immune cells [60] or low oxygen environment [61,62], which could exert completely divergent effects to shape the trophoblasts’ biological property (invasiveness) and thus, the overall growth trajectory of the fetus. In addition, the discrepancies seen among these reports with respect to in vivo growth responses could be due to the impact of the length (GD6-18 vs GD11-20), exposure regimen (continuous vs. intermittent), and mode (liquid or vapor chamber) of exposure of E. To unequivocally demonstrate the role of NRF2 dysregulation on in utero E on growth phenotype, it will thus be necessary to test this on NRF2 knockouts.

PCNA and CYCLIN-D1 govern the cellular proliferation and growth by coordinately regulating the cell cycle with DNA replication, where PCNA forms a DNA sliding clamp for replicative DNA polymerase and regulates DNA synthesis. CYCLIN-D1 regulates the G1/S-phase transition of the cell cycle [63]. Shown here, the 48 h exposure of human trophoblasts to E in vitro and in utero in rats resulted in a reduction of PCNA and CYCLIN-D1 expression (Figure 6 and Figure 7). Abridged PCNA and CYCLIN-D1 levels have been previously shown to impair the placental development in conditions that are underscored by oxidative stress akin to E [64,65]. While E-induced diminution of proliferation-associated proteins such as PCNA and CYCLIN-D1 which closely correlated with a reduction in the proliferative capacity in vitro, this was not reflected in the in utero E exposed rats with the unchanged placental and body weights (Figure 2). Based on these observations with the current IEV paradigm, we propose that concerted signal(s) other than PCNA and CYCLIN-D1 driven by spatial location (local environment) may be required to effect a gross change in the placental and overall growth. Nevertheless, a priming anti-growth program is established with respect to molecular changes (PCNA and CYCLIN-D1 levels) in the placenta. Relevantly, a complicated role for PCNA during the perinatal stage has been reported, where the downregulation of PCNA in mouse ovaries during primordial follicle formation, has been shown to favor the survival of oocytes [66]. While the current results do not completely exclude the ability of E to impact the proliferative or replicative strength of the trophoblast population. Especially in vivo, the complexities of post-translational modifications such as phosphorylation or ubiquitination and translocation of other key effectors in conjunction with PCNA and CYCLIN-D1 to modulate trophoblast-specific in vivo proliferative changes are being evaluated in our laboratory.

Finally, in vitro experiments using human trophoblasts demonstrated that *E* resulted in abnormal accumulation of p21, which correlated with proliferation inhibition (Figure 6). The p21 increase could be attributed to PCNA loss, since, PCNA has been shown to physically interact with and promote the degradation of p21 to switch on the DNA replication and cell cycle [67]. However, the intriguing finding is that the augmentation of p21 showed no-correlation with placental or gross body weight changes (representative of growth) when pregnant rats were challenged with intrauterine IEV exposure (Figure 2 and Figure 7). It is plausible that in the presence of a teratogen like E, despite high levels of p21, its localization could be altered due to phosphorylation by protein kinases, which curbs the p21′s negative cell cycle regulatory function. In particular, phosphorylation of p21 at Thr57 by glycogen synthase kinase 3β (GSK3β) has been shown to increase its ability to bind to cyclin B1-CDK1 complexes without inhibiting the complexes or cell-cycle progression, denoting the active growth process [68]. In a brain developmental setting, E has been shown to activate GSK3β [69]. Despite high levels of p21, its exclusion from the nucleus to cytosol has also been illustrated to favor a pro-growth shift [68]. It is also reasonable to argue that always anchoring the molecular expression pattern in dose, as well as in time and phenotypic outcome could be complex, as observed here with a non-linear pattern between E-elicited molecular changes and growth assessed in terms of body or placental weight. Nevertheless, our findings could suggest that the inductive molecular and cellular signaling change could occur well before the onset of defined cellular or biological phenotype. This result is particularly relevant to the developmental context since it is most often associated with producing patterns rather than traits. Thus, our results also draw attention to considering clusters of genes that regulate specific functions together with the cellular context, dose-response relationships, endogenous metabolism, molecular regulations via phosphorylations and protein-protein interactions.

## 5. Conclusions

In summary, the present report demonstrates repression of NRF2 in parallel with dysregulation of a PCNA/CYCLIN-D1/p21 growth-signaling network in E-exposed human trophoblast cells as well as in the placenta following second-trimester human equivalent intrauterine exposure to maternal IEV. While E-induced signaling alterations are functionally reflected in suppressing the proliferative ability in vitro, the in vivo molecular changes do not appear to grossly reflect on the weight-related growth of the placenta or the animal, per se. This could reflect compensatory systems active in the presence of normal maternal nutrition. Also, scientifically important findings here are the E-related dysregulation of NRF2 and its transactivation function observed both in vivo and in the human trophoblast model and the ability of the overexpression of NRF2 rendering (but only partial) recovery of the E-induced dysregulation of growth-promoting factors and cell survival of human trophoblasts (Figure 8).

Given all the molecular changes studied here are similar in both in vitro and in vivo setting, ethanol by dysregulating the NRF2-based cell stress pathway appears to have developed a primed or sensitized maladaptive profile that may alter the program of cell fate commitment and contribute to placental-associated complications.

## Figures and Tables

**Table 1:** List of Taqman gene primer-probe.

Gene	Assay ID	Fluorophore
*Gclc*	Rn00689046_m1	FAM
*Gclm*	Rn00568900_m1	FAM
*Gsr*	Rn01482159_m1	FAM
*Ho-1*	Rn01536933_m1	FAM
*Nqo1*	Rn00566528_m1	FAM
*Nfe2l2*	Rn00477784_m1	FAM
*Gapdh*	Rn01775763_g1	VIC

## References

[1] Burd J., Blair J., Dropps K. (2012). Prenatal Alcohol Exposure, Blood Alcohol Concentrations and Alcohol Elimination Rates for the Mother, Fetus and Newborn. J. Perinatol..

[2] Bailey B.A., Sokol J. (2008). Pregnancy and Alcohol Use: Evidence and Recommendations for Prenatal Care. J. Clin. Gynecol. Obstet..

[3] Jones K.L., Smith D.L., Ulleland C.W., Streissguth A.P. (1973). Pattern of Malformation in Offspring of Chronic Alcoholics. Lancet.

[4] Sulik K.K. (2014). Fetal Alcohol Spectrum Disorder: Pathogenesis and Mechanisms. Handb. Clin. Neurol..

[5] Clarren S.K., Smith D.W. (1978). The Fetal Alcohol Syndrome. N. Engl. J. Med..

[6] Jones K.L. (2011). The Effects of Alcohol on Fetal Development. Birth Defects Res..

[7] Henderson G.I., Schenker S. (1977). The Effects of Maternal Alcohol Consumption on The Viability and Visceral Development of The Newborn Rat. Res. Commun. Chem. Pathol. Pharmacol..

[8] Randall C.L., Taylor W.J. (1979). Prenatal Ethanol Exposure in Mice: Teratogenic Effects. Teratology.

[9] Patwardhan R.V., Schenker S., Henderson G.I., Abou-Mourad N.N., Hoyumpa A.M. (1981). Short-Term and Long-Term Ethanol Administration Inhibits the Placental Uptake and Transport of Valine in Rats. J. Lab. Clin. Med..

[10] Henderson G.I., Patwardhan R.V., McLeroy S., Schenker S. (1982). Inhibition of Placental Amino Acid Uptake in Rats Following Acute and Chronic Ethanol Exposure. Alcohol. Clin. Exp. Res..

[11] Aliyu M.H., Lynch O., Nana P.N., Alio A.P., Wilson R.E., Marty P.J., Zoorob R., Salihu H.M. (2011). Alcohol Consumption During Pregnancy and Risk of Placental Abruption and Placenta Previa. Matern. Child Health J..

[12] Gundogan F., Gilligan J., Qi W., Chen E., Naram R., de la Monte S.M. (2015). Dose Effect of Gestational Ethanol Exposure on Placentation and Fetal Growth. Placenta.

[13] Gundogan F., Elwood G., Mark P., Feijoo A., Longato L., Tong M., de la Monte S.M. (2010). Ethanol-Induced Oxidative Stress and Mitochondrial Dysfunction in Rat Placenta: Relevance to Pregnancy Loss. Alcohol. Clin. Exp. Res..

[14] Lane R.H., Ramirez R.J., Tsirka A.E., Kloesz J.L., McLaughlin M.K., Gruetzmacher E.M., Devaskar S.U. (2001). Uteroplacental Insufficiency Lowers the Threshold towards Hypoxia-Induced Cerebral Apoptosis in Growth-Retarded Fetal Rats. Brain Res..

[15] Basilious A., Yager J., Fehlings M.G. (2015). Neurological Outcomes of Animal Models of Uterine Artery Ligation and Relevance to Human Intrauterine Growth Restriction: A systematic review. Dev. Med. Child Neurol..

[16] Goeden N., Velasquez J., Arnold K.A., Chan Y., Lund B.T., Anderson G.M., Bonnin A. (2016). Maternal Inflammation Disrupts Fetal Neurodevelopment Via Increased Placental Output of Serotonin to the Fetal Brain. J. Neurosci..

[17] Kodomari I., Wada E., Nakamura S., Wada K. (2009). Maternal Supply of BDNF to Mouse Fetal Brain Through the Placenta. Neurochem. Int..

[18] Brunton P.J., Russell J.A. (2010). Endocrine Induced Changes in Brain Function During Pregnancy. Brain Res..

[19] Burton G.J., Fowden A.L. (2012). Review: The Placenta and Developmental Programming: Balancing Fetal Nutrient Demands with Maternal Resource Allocation. Placenta.

[20] Narasimhan M., Mahimainathan L., Rathinam M.L., Riar A.K., Henderson G.I. (2011). Overexpression of Nrf2 Protects Cerebral Cortical Neurons from Ethanol-Induced Apoptotic Death. Mol. Pharmacol..

[21] Patel D., Rathinam M., Jarvis C., Mahimainathan L., Henderson G., Narasimhan M. (2018). Role for Cystathionine γ Lyase (CSE) in an Ethanol (E)-Induced Lesion in Fetal Brain GSH Homeostasis. Int. J. Mol. Sci..

[22] Bosco C., Preedy V.R., Watson R.R. (2005). Alcohol and Xenobiotics in Placenta Damage. Compr. Handb. Alcohol Relat. Pathol..

[23] Rosenberg M.J., Wolff C.R., El-Emawy A., Staples M.C., Perrone-Bizzozero N.I., Savage D.D. (2010). Effects of Moderate Drinking During Pregnancy on Placental Gene Expression. Alcohol.

[24] De La Monte S.M., Wands J.R. (2001). Mitochondrial DNA Damage and Impaired Mitochondrial Function Contribute to Apoptosis of Insulin-Stimulated Ethanol-Exposed Neuronal Cells. Alcohol. Clin. Exp. Res..

[25] Lyall F. (2003). Development of The Utero-Placental Circulation: The Role of Carbon Monoxide and Nitric Oxide in Trophoblast Invasion and Spiral Artery Transformation. Microsc. Res. Tech..

[26] Schneider H. (2011). Oxygenation of the Placental-Fetal Unit in Humans. Respir. Physiol. Neurobiol..

[27] Myatt L. (2010). Review: Reactive Oxygen and Nitrogen Species and Functional Adaptation of the Placenta. Placenta.

[28] Baczyk D., Audette M.C., Coyaud E., Raught B., Kingdom J.C. (2018). Spatiotemporal Distribution of Small Ubiquitin-Like Modifiers During Human Placental Development and in Response to Oxidative and Inflammatory Stress. J. Physiol..

[29] Kay H.H., Grindle K.M., Magness R.R. (2000). Ethanol Exposure Induces Oxidative Stress and Impairs Nitric Oxide Availability in the Human Placental Villi: A Possible Mechanism of Toxicity. Am. J. Obstet. Gynecol..

[30] Devi B.G., Henderson G.I., Frosto T.A., Schenker S. (1993). Effect of Ethanol on Mitochondria Morphologic and Biochemical Integrity of The Fetal Rat Hepatocytes in Culture. Hepatology.

[31] Chen J., Robinson N.C., Schenker S., Frosto T.A., Henderson G.I. (1999). Formation of Hydroxynonenal Adducts with Cytochrome c Oxidase in Rats Following Short-Term Ethanol Intake. Hepatology.

[32] Chen J., Petersen D.R., Schenker S., Henderson G.I. (2000). Formation of Malondialdehyde Adducts in Livers of Rats Exposed to Ethanol: Role in Ethanol-Mediated Inhibition of Cytochrome c Oxidase. Alcohol. Clin. Exp. Res..

[33] Ma Q. (2013). Role of Nrf2 in Oxidative Stress and Toxicity. Annu. Rev. Pharmacol. Toxicol..

[34] Gong P., Cederbaum A.I. (2006). Nrf2 is Increased by CYP2E1 in Rodent Liver and Hepg2 Cells and Protects Against Oxidative Stress Caused by CYP2E1. Hepatology.

[35] Dong J., Sulik K.K., Chen S.Y. (2008). Nrf2-Mediated Transcriptional Induction of Antioxidant Response in Mouse Embryos Exposed to Ethanol *In vivo*: Implications for The Prevention of Fetal Alcohol Spectrum Disorders. Antioxid. Redox Signal..

[36] Kumar A., Singh C.K., Lavoie H.A., Dipette D.J., Singh U.S. (2011). Resveratrol Restores Nrf2 Level and Prevents Ethanol-Induced Toxic Effects in The Cerebellum of a Rodent Model of Fetal Alcohol Spectrum Disorders. Mol. Pharmacol..

[37] Bass T., Volpe J.J. (1989). Ethanol in Clinically Relevant Concentrations Enhances Expression of Oligodendroglial Differentiation but Has No Effect on Astrocytic Differentiation or DNA Synthesis in Primary Cultures. Dev. Neurosci..

[38] Riar A.K., Narasimhan M., Rathinam M.L., Vedpathak D., Mummidi S., Henderson G.I., Mahimainathan L. (2014). Ethanol-Induced Transcriptional Activation of Programmed Cell Death 4 (Pdcd4) Is Mediated by GSK-3β Signaling in Rat Cortical Neuroblasts. PLoS ONE.

[39] Delbridge L.M., Connell P.J., Harris P.J., Morgan T.O. (2000). Ethanol Effects on Cardiomyocyte Contractility. Clin. Sci..

[40] Toivari M., Mäki T., Suutarla S., Eklund K.K. (2000). Ethanol Inhibits Ige-Induced Degranulation and Cytokine Production in Cultured Mouse and Human Mast Cells. Life Sci..

[41] Narasimhan M., Rathinam M., Riar A., Patel D., Mummidi S., Yang H.S., Colburn N.H., Henderson G.I., Mahimainathan L. (2013). Programmed Cell Death 4 (PDCD4): A Novel Player in Ethanol-Mediated Suppression of Protein Translation in Primary Cortical Neurons and Developing Cerebral Cortex. Alcohol. Clin. Exp. Res..

[42] Riar A.K., Narasimhan M., Rathinam M.L., Henderson G.I., Mahimainathan L. (2016). Ethanol Induces Cytostasis of Cortical Basal Progenitors. J. Biomed. Sci..

[43] Graham C.H., Hawley T.S., Hawley R.G., MacDougall J.R., Kerbel R.S., Khoo N., Lala P.K. (1993). Establishment and Characterization of First Trimester Human Trophoblast Cells with Extended Lifespan. Exp. Cell Res..

[44] Kilburn B.A., Wang J., Duniec-Dmuchowski Z.M., Leach R.E., Romero R., Armant D.R. (2000). Extracellular Matrix Composition and Hypoxia Regulate the Expression of HLA-G and Integrins in a Human Trophoblast Cell Line. Biol. Reprod..

[45] Patten A.R., Fontaine C.J., Christie B.R. (2014). A Comparison of the Different Animal Models of Fetal Alcohol Spectrum Disorders and their Use in Studying Complex Behaviors. Front. Pediatr..

[46] Rice D., Barone S. (2000). Critical Periods of Vulnerability for the Developing Nervous System: Evidence from Humans and Animal Models. Environ. Health Perspect..

[47] Wong C.T., Wais J., Crawford D.A. (2015). Prenatal Exposure to Common Environmental Factors Affects Brain Lipids and Increases Risk of Developing Autism Spectrum Disorders. Eur. J. Neurosci..

[48] Adamson S.L., Lu Y., Whiteley K.J., Holmyard D., Hemberger M., Pfarrer C., Cross J.C. (2002). Interactions Between Trophoblast Cells and the Maternal and Fetal Circulation in the Mouse Placenta. Dev. Biol..

[49] Gilpin N.W., Richardson H.N., Cole M., Koob G.F. (2008). Vapor Inhalation of Alcohol in Rats. Curr. Protoc. Neurosci..

[50] Morton R.A., Diaz M.R., Topper L.A., Valenzuela C.F. (2014). Construction of Vapor Chambers used to Expose Mice to Alcohol During the Equivalent of all Three Trimesters of Human Development. J. Vis. Exp..

[51] Narasimhan M., Patel D., Vedpathak D., Rathinam M., Henderson G., Mahimainathan L. (2012). Identification of Novel MicroRNAs in Post-Transcriptional Control of Nrf2 Expression and Redox Homeostasis in Neuronal, SH-SY5Y Cells. PLoS ONE.

[52] Narasimhan M., Riar A.K., Rathinam M.L., Vedpathak D., Henderson G., Mahimainathan L. (2014). Hydrogen Peroxide Responsive Mir153 Targets Nrf2/ARE Cytoprotection in Paraquat Induced Dopaminergic Neurotoxicity. Toxicol. Lett..

[53] Dimauro I., Pearson T., Caporossi D., Jackson M.J. (2012). A Simple Protocol for the Subcellular Fractionation of Skeletal Muscle Cells and Tissue. BMC Res. Notes.

[54] Morabito C., Guarnieri S., Catizone A., Schiraldi C., Ricci G., Mariggiò M.A. (2017). Transient Increases in Intracellular Calcium and Reactive Oxygen Species Levels in Tcam-2 Cells Exposed to Microgravity. Sci. Rep..

[55] Kay H.H., Tsoi S., Grindle K., Magness R.R. (2006). Markers of Oxidative Stress in Placental Villi Exposed to Ethanol. J. Soc. Gynecol. Investig..

[56] Rodrigo S., Rodríguez L., Otero P., Panadero M.I., García A., Barbas C., Roglans N., Ramos S., Goya L., Laguna J.C. (2016). Fructose During Pregnancy Provokes Fetal Oxidative Stress: The Key Role of The Placental Heme Oxygenase-1. Mol. Nutr. Food Res..

[57] Zhao F., Lei F., Yan X., Zhang S., Wang W., Zheng Y. (2018). Protective Effects of Hydrogen Sulfide against Cigarette Smoke Exposure-Induced Placental Oxidative Damage by Alleviating Redox Imbalance Via Nrf2 Pathway in Rats. Cell. Physiol. Biochem..

[58] Lee H.M., Choi K.C. (2018). Cigarette Smoke Extract and Isoprene Resulted in the Induction of Apoptosis and Autophagy in Human Placenta Choriocarcinoma JEG-3 Cells. Environ. Toxicol..

[59] Gu H., Li H., Zhang L., Luan H., Huang T., Wang L., Fan Y., Zhang Y., Liu X., Wang W. (2012). Diagnostic Role of MicroRNA Expression Profile in the Serum of Pregnant Women with Fetuses with Neural Tube Defects. J. Neurochem..

[60] Erlebacher A. (2013). Immunology of the Maternal-Fetal Interface. Annu. Rev. Immunol..

[61] Genbacev O., Joslin R., Damsky C.H., Polliotti B.M., Fisher S.J. (1996). Hypoxia Alters Early Gestation Human Cytotrophoblast Differentiation/Invasion In vitro and Models the Placental Defects that Occur in Preeclampsia. J. Clin. Investig..

[62] Rosario G.X., Konno T., Soares M.J. (2008). Maternal Hypoxia Activates Endovascular Trophoblast Cell Invasion. Dev. Biol..

[63] Sourisseau T., Georgiadis A., Tsapara A., Ali R.R., Pestell R., Matter K., Balda M.S. (2006). Regulation of PCNA and Cyclin D1 Expression and Epithelial Morphogenesis by the ZO-1-Regulated Transcription Factor ZONAB/DbpA. Mol. Cell. Biol..

[64] Cao X., Hua X., Wang X., Chen L. (2017). Exposure of Pregnant Mice to Triclosan Impairs Placental Development and Nutrient Transport. Sci. Rep..

[65] Banu S.K., Stanley J.A., Sivakumar K.K., Taylor R.J., Arosh J.A., Burghardt R.C. (2017). Editor’s Highlight: Exposure to CrVI during Early Pregnancy Increases Oxidative Stress and Disrupts the Expression of Antioxidant Proteins in Placental Compartments. Toxicol. Sci..

[66] Xu B., Hua J., Zhang Y., Jiang X., Zhang H., Ma T., Zheng W., Sun R., Shen W., Sha J. (2011). Proliferating Cell Nuclear Antigen (PCNA) Regulates Primordial Follicle Assembly by Promoting Apoptosis of Oocytes in Fetal and Neonatal Mouse Ovaries. PLoS ONE.

[67] Abbas T., Sivaprasad U., Terai K., Amador V., Pagano M., Dutta A. (2008). PCNA-Dependent Regulation of P21 Ubiquitylation and Degradation Via the CRL4Cdt2 Ubiquitin Ligase Complex. Genes Dev..

[68] Besson A., Dowdy S.F., Roberts J.M. (2008). CDK Inhibitors: Cell Cycle Regulators and Beyond. Dev. Cell..

[69] Liu Y., Chen G., Ma C., Bower K.A., Xu M., Fan Z., Shi X., Ke Z.J., Luo J. (2009). Overexpression of Glycogen Synthase Kinase 3beta Sensitizes Neuronal Cells to Ethanol Toxicity. J. Neurosci. Res..

